# The Ecological Assessment of Responses to Speaking-up tool—development and reliability testing of a method for coding safety listening behavior in naturalistic conversations

**DOI:** 10.3389/fpubh.2025.1652250

**Published:** 2025-10-14

**Authors:** Alyssa M. Pandolfo, Tom W. Reader, Alex Gillespie

**Affiliations:** ^1^Department of Psychological and Behavioural Science, London School of Economics and Political Science, London, United Kingdom; ^2^Oslo New University College, Oslo, Norway

**Keywords:** safety listening, safety voice, coding framework, safety communication, coding tool, behavioural marker system

## Abstract

**Introduction:**

Safety communication is crucial for accident aversion across industries. While researchers often focus on encouraging concern-raising (‘safety voice’), responses to these concerns (‘safety listening’) remain underexplored. Existing studies primarily use self-report measures; however, these tend to focus on perceptions of listening rather than behaviors. To fully understand and examine how safety listening is enacted and influential in safety-critical environments, a tool for reliably assessing naturalistic safety listening behaviors in high-risk settings is required. Accordingly, we developed and tested the Ecological Assessment of Responses to Speaking-up (EARS) tool to code safety listening behaviors in flightdeck conversations.

**Methods:**

There were three analysis phases: (1) developing the taxonomy through a qualitative content analysis (*n* = 45 transcripts); (2) evaluating interrater reliability and coder feedback (*n* = 40 transcripts); and (3) testing the taxonomy’s interrater reliability in a larger unseen dataset (*n* = 110 transcripts) and with an additional coder (*n* = 50 transcripts).

**Results:**

Contrary to the notion that effective listening is agreement, our findings emphasize engagement with safety voice, including reasonable disagreement. The final taxonomy identifies six safety listening behaviors: action (implementing, declining), sensemaking (questioning, elaborating), and non-engagement (dismissing, token listening) and two additional voice acts (escalating, amplifying). EARS achieved substantial interrater reliability (Krippendorff’s alpha of 0.73 to 0.77 and Gwet’s ACT1 of 0.80 to 0.87).

**Discussion:**

The EARS tool allows researchers to assess safety listening in naturalistic conversations, facilitating analysis of its antecedents, its interplay with safety voice, and the impact of interventions on outcomes.

## 1 Introduction

Effectively raising and responding to safety concerns is critical for averting organizational failures. Accidents like the Challenger and Columbia space shuttle disasters, the Boeing 737 MAX crashes, and the Deepwater Horizon catastrophe have all highlighted how problems in speaking up *and* responding effectively to voice have contributed to incidents (1–3). Consequently, safety researchers have extensively focused on how organizations can encourage individuals to speak up with safety concerns (‘safety voice’), establishing team- and institutional-level voice antecedents and integrating effective safety voice behaviors into behavioral marker systems (4, 5). Yet, while safety voice is often crucial for maintaining safety and preventing accidents, it cannot achieve this without ‘safety listening’, which relates to how listeners engage with the content of safety voice (6). As illustrated in Table 1, safety listening is an important factor in both causing and preventing accidents. In these instances, individuals raised concerns about safety problems, and the response of individuals or organizations to these concerns (e.g., engaging with or dismissing issues) determined the outcomes.

**Table 1:** Example case studies illustrating listening behaviors.

Case	Year(s)	Example voice act	Example listening act	Outcome	Interpretation of listener behavior
1. South African DC-40 (69)	1962	An air traffic controller noticed that a South African DC-40 aircraft was on a collision course with another aircraft. They instructed, ‘Descend to 35 [i.e., 3,500’] now’.	The DC-40’s cockpit responded, ‘Descending to 35 [i.e., 3,500’] now’.	The DC-40 did not descend despite verbally agreeing to and collided with the other aircraft shortly after.	Listening is not necessarily verbally agreeing with instructions; listeners’ actions must match their words.
2. Johnson and Johnson Tylenol Tampering (70)	1982	A Chicago news reporter called Johnson and Johnson, saying that a medical examiner had given a press conference about seven people dying from poisoned Tylenol.	Johnson and Johnson alerted customers not to consume Tylenol and withdrew all Tylenol capsules from Chicago stores. After finding two contaminated bottles, they ordered a national recall.	An unknown individual had tampered with and laced Tylenol capsules with cyanide. Johnson and Johnson developed tamper-resistant packaging and capsules.	Listening is pausing the status quo and investigating the problem.
3. US Airways 1549 (71)	2009	The airplane struck birds shortly after take-off. The air traffic controller suggested returning to the originating airport, suggesting, ‘if we can get it for you do you want to try to land runway 13?’	The captain declined, saying, ‘We’re unable. We may end up in the Hudson [River]’.	The cockpit crew landed the aircraft in the Hudson River with no fatalities.	Listening is disagreeing with infeasible voiced requests.
4. Grenfell Tower Fire (72)	2017	In a public blog, a Grenfell Tower resident wrote that, ‘only a catastrophic event will expose the ineptitude and incompetence of our landlord […] and bring an end to the dangerous living conditions and neglect of health and safety legislation that they inflict upon their tenants and leaseholders’.	The tenant management organization did not respond to or investigate the blog’s claims.	Six months after the blog post, a fire blazed through Grenfell Tower, burning for 60 h and killing 72.	Listeners did not respond to the complaint. This can be classified as no response, ignoring, and/or inaction.
5. Hawaii False Missile Alert (73)	2018	A Hawaii Emergency Management Agency employee mistakenly issued a public alert warning during a routine drill. Hawaiians received a message stating, ‘Ballistic missile threat inbound to Hawaii. Seek immediate shelter. This is not a drill’.	Colleagues recognized the error and issued a correction 38 min after the initial alert.	While the false alarm caused panic, there was no physical harm reported.	Voicers may be misguided—this employee genuinely believed there was an impending missile attack. Here, listening was correcting misperceptions.
6. Delta Air Lines Flight 1943 (74)	2023	An air traffic controller at John F. Kennedy Airport noticed that Delta 1943 was taking off while another aircraft was crossing the runway. They voiced: ‘Shit! Delta 1943, cancel take-off clearance’.	The cockpit crew responded, ‘Rejecting. Alright, oof, Delta 1943’, confirming that they were stopping the aircraft.	The airplanes did not collide.	Listening is a verbal agreement with voice and confirmation of actions.

Despite the apparent importance of safety listening for preventing accidents and improving safety, the literature lacks an established method for assessing listening behaviors. Safety listening measures are typically self-reports, such as surveys and interviews (7–9). While self-report measures these provide insights into experiences or perceptions of listening within organizations, they do not support the evaluation and improvement of safety listening. Behavioral marker systems research has shown how structured and theory-based assessments of live behavior in safety–critical environments are essential for documenting different forms of behavior that link to outcomes, supporting training, evaluating interventions, and changing organizational culture (4). For example, behavioral marker systems have been extensively used to improve teamwork and decision-making in healthcare (10). Hitherto, when safety listening has been studied naturalistically or through observational frameworks, researchers have variously conceptualized and operationalized the categories of behavior that comprise listening (11–14).

To understand how exactly safety listening shapes outcomes in dynamic and high-risk contexts, and investigate the effectiveness of listening behavior in teams, a reliable method of assessing safety listening behaviors in naturalistic high-stakes conversations is needed. This method would enable safety researchers to analyze live behavior and novel behavioral datasets, improve findings’ comparability, support assessment and training, and evaluate listening before and after interventions. Here, we develop and test the Ecological Assessment of Responses to Speaking-up (‘EARS’) tool and test its reliability by using it to classify instances of safety listening in transcripts of flight crews interacting during safety incidents.

### 1.1 Background

Communication about hazards is a necessary precursor to promoting safety and averting organizational failures. For example, Westrum’s (15) theory of information flow argues that communication on risks, events, and anomalies is essential for ensuring that hazards are understood and effectively managed. Conversely, communication breakdowns (e.g., due to silos, scapegoating of those who share negative information) impair safety management (15). As safety communication is ultimately anchored in individuals sharing information, researchers have extensively investigated behaviors underlying safety voice (raising concerns about safety hazards), exploring how speaking-up can be encouraged in organizations (5, 16). Through this work, researchers have established a conceptualization of safety voice (17), with speaking-up viewed as a spectrum between direct communication and silence (18), and constructs like ‘muted voice’ capturing indirect forms of safety voice where individuals hint at rather than push concerns (19). Academics and practitioners have typically assessed safety voice using self-report measures (5); however, more recent studies have identified observable voice behaviors (e.g., expressing concerns) and have measured them in naturalistic settings (12, 13).

In contrast to the literature’s focus on safety voice, fewer studies have explored its counterpart: safety listening. Academics have variously conceptualized safety listening within and between literatures, with a recent conceptual review finding 36 unique terms/definitions (6). While some neutral terminologies were used [e.g., ‘receiver response’; (8)], most depicted consequences of voicing [e.g., ‘retaliation’; (20)]. Likewise, listening was framed as motivational via terms like ‘willful blindness’ (21) and the ‘deaf ear syndrome’ (22) and responses were explained as strategic maneuvers within games [e.g., ‘blame games’ and ‘organizational jiu-jitsu’; (23, 24)]. In sum, the literature has generally viewed safety listening as listeners’ attitudes following voice, with poor responses positioned as motivational and deliberate.

Synthesizing the literature’s conceptualizations, Pandolfo et al. (6) conclude that safety listening is more usefully defined as a listeners’ responses to speaking up, requesting action to prevent harm. This definition positions safety listening as behavioral rather than attitudinal: it is how listeners act to understand or address potential hazards, with attitude being among various factors (e.g., training, knowledge and skills, clarity of voice act) that shape whether and how listeners respond to voice effectively. Listening is conceptualized in terms of the distinct patterns of behavior—for instance, approving, ignoring, replying, retaliating—that occur after speaking-up (25). Accordingly, the effectiveness of safety listening is determined by whether the response engages with the voice act. Consequently, discounting safety voice may be effective if it is suitable (e.g., voicers raised incorrect information), respectful, does not hinder future voice acts, and does not cause harm. As illustrated in Table 1, adopting a behavioral approach to safety listening is important because how listeners respond to voice can vary according to the situation being faced, and with responses enabling the individual and collective sensemaking required to address problems and prevent harm (26). Where listening behaviors are ineffective—for instance, dismissing legitimate concerns and/or acting on safety voice, which is well-motivated but incorrect—understanding and decision-making on risk is impaired. Hitherto, despite the importance of safety listening in averting accidents, there is currently no established method of reliably assessing safety listening in high-risk settings.

### 1.2 The need for a safety listening behavior assessment

As discussed above, research in the domain of safety has rarely taken a behavioral approach to investigate safety listening, despite listeners’ behaviors being self-evidently crucial for preventing accidents. Where research has been done, it has generally used surveys and interviews, rather than ‘live’ investigations of natural behavior (6). Using proxies of behavior resonates with more general critiques of psychological research, where there has been a push for psychologists to measure naturalistic behavior to ensure that observations are grounded in real and consequential activity (27, 28).

Developing a method for assessing safety listening in natural and safety–critical settings would advance theory and practice. Pandolfo et al. (6), conceptual review found that two out of 46 empirical safety listening studies assessed naturalistic behaviors; the remainder employed self-report methods or assessed hypothetical behaviors, with these being used as proxies for naturalistic behavior. While valuable for understanding safety listening, such proxies can have critical limitations—most importantly, it cannot be assumed that findings in safe and created settings generalize to uncertain environments with potential harms, or will effectively predict and explain how individuals will respond to safety voice in complex and uncertain situations (29). Self-reported listening intentions and behaviors may be inaccurately described, recalled, and attributed due to biases (e.g., social desirability), errors (e.g., memory errors), and misinterpretations (30, 31). Researchers also have studied generalized responses (e.g., whether individuals feel listened to on average) rather than the actual moments of listening that have direct consequences for outcomes (e.g., engaging with concerns). Hypothetical vignettes may over-rely on participants’ imaginations, while experiments require participants’ belief in confederates’ roles and instructions (32). Moreover, assessing voicers’ and listeners’ perceptions of listening may not correlate with behavioral listening assessments, as Bodie et al. (33) found no association between these three measures. Likewise, Collins (34) argues that verbal markers (e.g., paraphrasing, follow-up questions) are the best listening indicators because they cannot be faked.

Another limitation of the safety listening literature’s focus on behavioral proxies is its tendency to frame safety communications as one-off exchanges (i.e., one voice act, one listening act, and then outcome) in clear-cut situations with defined speaker roles and actionable concerns (6). For instance, Long et al. (8) posit that after safety voice, listeners determine and enact their response, and their response has implications for patient care and team dynamics. Yet, naturalistic safety conversations are not always one-shot and unambiguous. For example, as the events of September 11, 2001 (coordinated terrorist hijackings targeting the World Trade Center and the Pentagon) were unfolding, Northeast Air Defense Sector members were initially misguided, incorrectly believing they were in a training simulation (35). Some members voiced doubt (*‘Is this real-world or exercise?’*), prompting further discussions, which improved the team’s understanding of the situation. Namely, the team first updated their belief to be that the hijacks were real but prototypical (*‘[the situation] will simmer down and we’ll probably get some better information [i.e., hijackers will make demands]’*) and then ultimately reached the correct conclusion that this was a coordinated attack (*‘if this stuff [i.e., multiple hijacks/attacks] is gonna keep on going, we need to take those fighters [fighter jets], put ‘em over Manhattan’*). Consequently, safety conversations may be an iterative process characterized by sensemaking, blurred delineations between voicers’ and listeners’ roles, and misguided interpretations of situations (6, 26).

Researchers’ use of self-report measures and experiments to study safety listening is understandable due to challenges with accessing—particularly in a controlled and rigorous manner—real-life situations where listening is critical for accident prevention. Recent advances in technology and the availability of digital data have made it increasingly possible to study safety listening through observational and/or behavioral trace data, enabling theory and empirical observations to be anchored in ecologically valid and contextualized situations [i.e., those that contain real safety threats; (36)]. While researchers always could study safety listening behaviors *in situ* [‘naturalistic observation’; e.g., (12)], they can now use new technologies like body cameras [‘ecological observation’; (37)] or behavioral trace data—transcripts or recordings of actual conversations containing safety voice and listening. Pandolfo et al. (6) give examples of unobtrusive and public datasets, including healthcare complaints and space mission communications.

To capitalize on these new opportunities and datasets, a framework of observable safety listening behaviors is required. Research on assessing and improving non-technical skills in safety–critical domains has long emphasized that, to reliably and meaningfully assess behavior, researchers must develop theoretically coherent taxonomies of observable behaviors that either enhance or impede safety performance (4). Studying safety listening through observations, therefore, is necessary to develop a framework of the core behaviors through which individuals respond to safety voice, establish the reliability of this framework, and then explore the link to outcomes. Such behaviors—according to the literature—may include various and quite basic categories like addressing problems, elaborating, agreeing with concerns, ignoring complaints, disagreeing with concerns, rejecting, or denigrating the voicer (12–14, 38). Furthermore, the impact of such behaviors upon safety outcomes is not necessarily straightforward and is challenging to evaluate outside of the context of the situation in which they occur. For instance, while the listening literature generally views disagreeing with voice as indicative of poor listening [and measures listening through whether voicers feel heard; (39)], effective safety listening can involve *not* acting on a voice (because it is misguided), interpreting a voice act as a signal of a more profound misunderstanding (e.g., on the goal of a team), or questioning the voicer to fully understand a problem (6).

In sum, there is minimal standardization of how to code naturalistic safety listening or to understand how these behaviors contribute to safety outcomes. Generating a standardized, reliable, and accurate classification system would have multiple benefits for the literature: it would enable the reliable coding of safety listening in naturalistic data (6), improve the conceptualization of safety listening and its impact on safety outcomes through anchoring it in contextualized behaviors (i.e., responses to voice acts) rather than general attitudes (40), and support practical efforts to assess and improve listening behaviors in organizations through evaluating how individuals respond to safety voice acts and findings’ comparability (4).

### 1.3 Current study

To address the above research gap, the current study reports on the development of the EARS tool for measuring safety listening through analyzing conversational dialog. The tool’s function is to support the reliable observation of safety listening in both live settings and transcript data. To build the tool, we focused on one of the most consequential and curatable data sources for studying safety listening: flightdeck conversations (36).

#### 1.3.1 Flightdeck conversations

Flightdeck conversations involve exchanges within teams regarding take-off and landing authorizations, weather updates (e.g., wind speeds), and issue reporting (e.g., flap problems). English is the standard language (77); however, local languages may be used in non-essential communications within cockpit teams.

Flightdeck conversations are generally accessed through cockpit voice recorders (CVRs) and/or air traffic control (ATC) radio recordings that document flightdeck interactions. CVRs capture all sounds—including alarms—and conversations within the cockpit, involving captains, first officers, and ATC. Conversely, ATC recordings capture communications and noises broadcast over specific radio channels but do not include intra-cockpit conversations. Globally, airports record ATC communications for purposes like accident analysis and training, and anyone within 15 miles/24 km of airports can also record these conversations (41).

These recordings are predominantly available through two sources. First, government agencies transcribe and publish CVR and ATC recordings in detailed incident reports. These reports are thorough and independently conducted, combining multiple sources of evidence to understand and learn from incidents. Second, volunteers often upload ATC recordings to platforms like YouTube to be used for educational purposes (e.g., assisting student pilots in learning aviation terminology). Table 2 shows a sample transcript.

**Table 2:** Sample flightdeck conversation transcript.

In 2017, Air China 428 (‘CCA428’) was climbing out of runway 25 L when the crew mistakenly turned left into the mountains of Lantau Island. The ATC noticed and instructed the pilots to turn immediately and expedite climb.				
Line	Speaker	Transcript	Code	Interpretation
1	Hong Kong Tower	Air China 428, line up and wait 25 L.		
2	CCA428	Line up and wait 25 L, Air China 428.		
3	Hong Kong Tower	Air China 428, wind 140 degrees 6 knots, runway 25 L, cleared for take-off.		
4	CCA428	Cleared for take-off, runway 25 L, Air China 428.		
5	Hong Kong Tower	Air China 428, Departure is 123.8; good day!		
6	CCA428	123.8, good day; Air China 428.		
7	Hong Kong Departure	Air China 428, Departure, identified. Climb FL130.		
8	Hong Kong Departure	Air China 117, contact Approach 119.1.		
9	Hong Kong Departure	Air China 428, cleared FL130.		
10	CCA428	Cleared FL130, Air China 428.		
11	Hong Kong Departure	Air China 428, climb FL130.		
12	Hong Kong Departure	Thai Asia 505, climb FL160.		
13	AIQ505	Climb FL160, Thai Asia 505.		
14	Hong Kong Departure	China Eastern 506, climb FL160.		
15	CES506	Climbing to FL160, China Eastern 506.		
16	Hong Kong Departure	Air China 428, turn right immediately. Turn right immediately. Heading 0—correction—heading 270. Terrain ahead. Expedite climb.	Safety voice	The ATC instructed CCA428 to turn right and climb immediately as there was terrain ahead.
17	Hong Kong Departure	Air China 428?		No response from CCA428; the ATC checked if they had heard.
18	CCA428	[Radio noises]		Unclear response from CCA428.
19	ATC	Air China 428, expedite climb. Terrain ahead—terrain alert! Expedite passing 5,000 feet. Expedite!	Escalating safety voice	
20	CCA428	Expedite, Air China 428.	Implementing	
21	CCA428	Air China 428 is now flying to RUMSY.	Incident averted	

We used CVR and ATC recordings to develop the EARS tool for the following reasons. First, researchers have encouraged the aviation industry to consider and train interpersonal skills within sociotechnical systems to enhance safety (42). Psychological safety, safety voice, and safety listening have been identified as crucial behaviors underpinning aviation safety, which sometimes require improvement (13, 43). For instance, out of 172 transcripts preceding aviation accidents, Noort et al. (13) identified 82 incidents which exclusively had effective listening and 33 that contained repeated poor listening. Thus, this dataset contains variability in safety listening types.

Second, flightdeck conversations are world-making (44). These recordings unobtrusively capture actual safety communication in risky situations, depicting how safety voice and listening behaviors cause or avert accidents. Aviation’s standardization (e.g., linguistic patterns, training, encountered risks) provides a controlled and ecologically valid setting to examine safety communication behaviors. Using this dataset follows recommendations to balance behavioral and self-report assessments in psychology (28) and avoids self-reports’ limitations (e.g., memory errors and attribution biases). In this study, we analyze flightdeck conversations before safety incidents, as these situations are where safety listening is most likely to be observable and consequential.

Third, aviation crew members understand that their conversations are recorded via CVRs, airport operational monitoring systems, and by individuals interested in aviation. Governmental agencies and aviation enthusiasts have made flightdeck conversations publicly available with the intent of explaining incidents and improving aviation safety; our use aligns with this intended purpose.

#### 1.3.2 Study aims

Using flightdeck conversations as our data source, we aimed to (1) develop and test the EARS tool and (2) explore its reliability in classifying safety listening and potential for informing organizational learning. We did this in three phases.

First, in taxonomy development, we used directed and summative content analysis to create the first version of EARS. This phase involved deductively applying a coding framework derived from the literature to 45 transcripts, inductively grouping similar behaviors and identifying listening forms not covered by the literature, and abductively exploring tensions between the literature and the transcripts.

Second, in taxonomy iteration, four coders applied the initial version of EARS to the same 10 transcripts. Interrater reliability and coder feedback were used to develop the next iteration of EARS, which was subsequently applied to a new batch of 10 transcripts. This process was repeated until we deemed saturation; this occurred after four rounds of iteration.

Third, in taxonomy testing, three coders coded 110 transcripts with the final version of EARS. A fourth coder, previously uninvolved with this study, coded 50 of the same transcripts using the final version. EARS obtained substantial interrater reliability in both analyses, and we investigated instances of coder disagreement.

When creating EARS, we drew upon the non-technical skills and behavioral marker systems literature. This literature posits that, in addition to developing technical skills (e.g., flight skills), assessing and training non-technical skills like teamwork, leadership, situation awareness, and decision-making is essential for safe and effective work performance (4). As previously discussed, behavioral marker systems—observational tools that taxonomize and assess non-technical skills—are widely used to identify and improve such behaviors in organizations (e.g., in surgery and aviation). Hitherto, while studies of non-technical skills often show communication as essential for success (e.g., in aviation), behavioral marker systems usually focus on behaviors like transmitting plans or delegating tasks rather than on listening behaviors (45).

Our study contributes to the literature by creating a behavioral marker system for safety listening: the EARS tool. This tool taxonomizes safety listening’s observable behaviors, and we test its reliability using flightdeck conversation transcripts. The EARS tool will enable research, contribute to theory development on voice and listening, and support practical interventions and efforts at organizational learning (e.g., in assessing the state and quality of safety listening within organizations). The tool aims to shift how we understand safety listening: moving it to a behavioral perspective that focuses more on whether listening happens (and its impact on safety) rather than attitudes and beliefs around listening which may or may not accurately reflect what occurs in specific and high-consequence safety situations.

## 2 Methods and results

**Figure 1:**
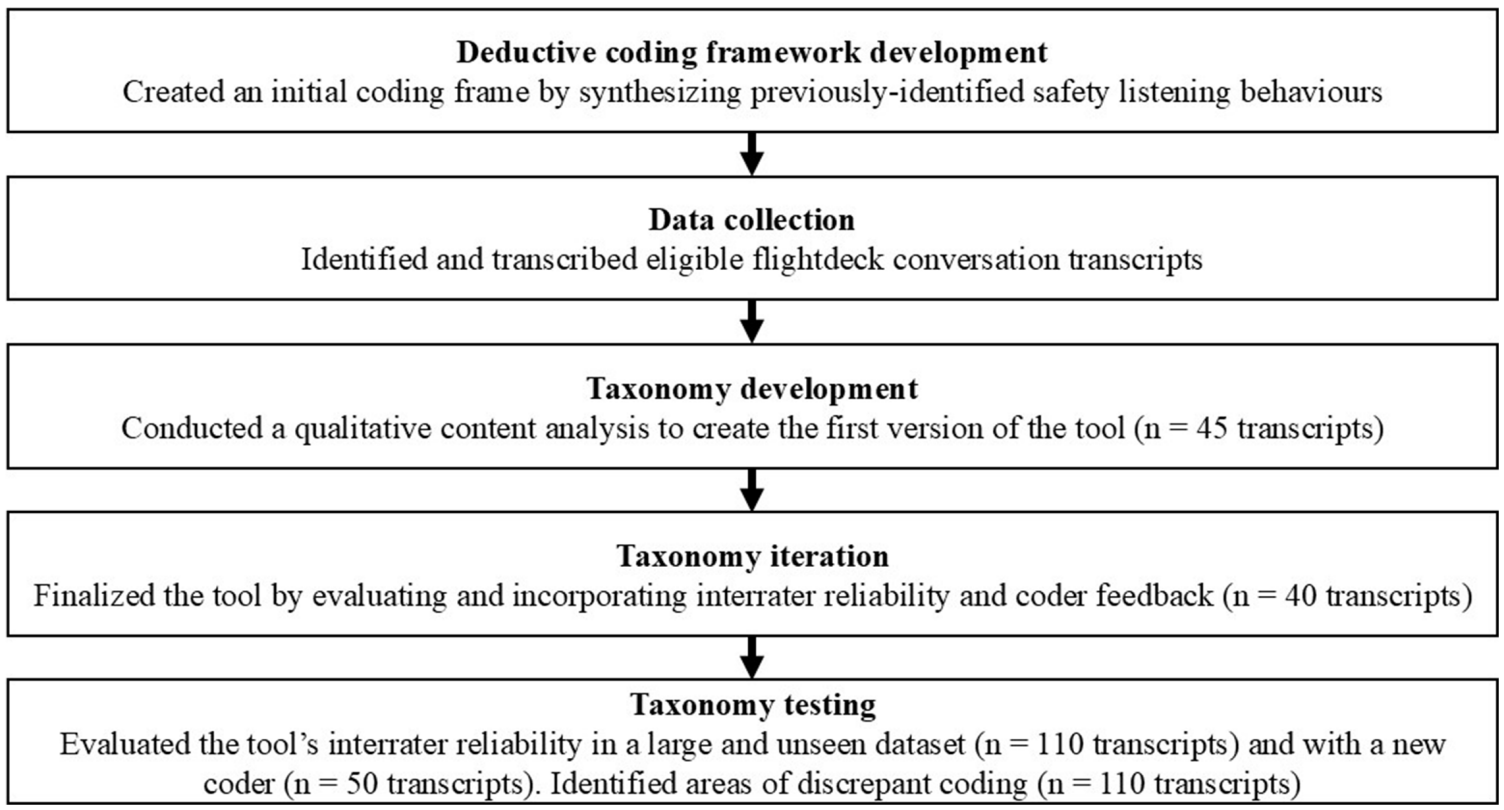
Process diagram.

We developed, iterated, and tested the EARS coding framework of safety listening behaviors, assessing listening at the turn level. All authors are research psychologists with experience analyzing flightdeck conversations and mixed-methods research. Authors 2 and 3 have developed and validated multiple psychological assessments. Figure 1 shows a process diagram summarizing the tool creation steps.

### 2.1 Deductive coding framework development

We first created an initial coding framework of safety listening types, informed by the literature. We searched for articles that identified and/or assessed different types of listening behavior. Specifically, we examined the 57 articles included in Pandolfo et al. (6) safety listening conceptual review, the 53 listening scales in Fontana et al.’s (46) systematic review, and relevant classifications from other literature, including sensemaking (47, 48), defensiveness (49) and voice cultivation (50).

Four articles classified and/or assessed safety listening behaviors, summarized in Table 3. Using abduction, we grouped similar behaviors, finding that safety listening responses were consistent with the voice request, inconsistent with the voice request, or did not respond to the voice. Table 4 shows our initial deductive coding frame, which we applied to the data and refined in the taxonomy development stage.

**Table 3:** Safety listening behavior measurements in the literature.

Authors	Method	Sample	Safety listening types	Safety listening measurement
Noort et al. (13)	Behavioral trace data	172 flightdeck conversations transcripts preceding aviation crashes between 1962 and 2018.	Ignored (0) Disaffirmed (−1) Verbally affirmed (1) Immediate action (2)	Safety listening was coded within three conversational turns after safety voice. Each instance of safety listening was assigned a score (in brackets beside each type), and the authors averaged each transcript’s safety listening score.
Lemke et al. (12)	Naturalistic observations	Approximately 12.5 h of *in situ* observations of anesthesia teams.	Verbal Short approval Elaboration Rejection No verbal or non-verbal reaction Behavioral Content is implemented Content is not implemented Affect Enthusiasm Interest Validation Contempt Defensiveness Fear/tension Neutral	The observer assigned and documented pre-defined behavioral safety listening codes during observations.
Reader (14)	Case study	The five-volume public inquiry for the Mid Staffordshire NHS Foundation Trust’s patient neglect scandal. It consists of interviews with 966 patients and relatives and 82 current and former staff members.	Accepted Denied Ignored Inaction	Textual excerpts were coded in terms of strategies for responding to safety voice using content analysis.
Barlow et al. (11)	Simulation	Twenty-two medical simulations involving a patient discharge. A confederate raised a scripted concern that the patient would be discharged with insufficient home care.	Emotional expression (e.g., providing reassurance) Interpersonal control (e.g., constraining others’ speaking up using power differentials) Interpretability (e.g., clarifying concerns)	Participants’ responses to the confederate’s concern were recorded and coded.

**Table 4:** Deductive coding framework.

Code	Description	Relevant terminologies	Example
Response consistent with voice	Speech and/or action consistent with the voice request	Immediate action, verbally affirmed (13); content is implemented, short approval, elaboration (12); accepted (14); sensemaking (47, 48); responding to speakers (46); asking questions (46)	*Air Canada 759* United 1: United 1, Air Canada flew directly over us. ATC: Yeah, I saw that, guys.
Response inconsistent with voice	Speech and/or action inconsistent with the voice request	Disaffirmed (13); denied (14); rejection (12); avoiding, delegitimizing, limiting tactics (49); avoiding, opposing (50)	*Horizon Air* ATC: We’re just trying to find a place for you to land safely. Hijacker: Yeah, I’m not quite ready to bring it [aircraft] down yet.
No response	No verbal or behavioral response following the voice	Ignored (13, 14); inaction (14); no verbal or non-verbal reaction, content not implemented (12); avoiding tactics (49); avoiding (50)	*Diamond N859PA* ATC: N859PA, this is Philly Approach. If you are on frequency, acknowledge. You’re entering a restricted flight area. You need to turn northeast bound to exit. ATC: N859PA, this is Philly Approach on Guard. If you can hear this transmission, acknowledge.

Reproduced from “Appendix: The deductive coding framework” Pandolfo, Reader, and Gillespie, under CC BY 4.0 (78).

### 2.2 Data collection

Our data were flightdeck conversations preceding near misses and crashes (collectively referred to as ‘incidents’). We obtained actual flightdeck conversation transcripts and recordings from five public data sources, summarized in Table 5. Author 1 reviewed the datasets and assessed cases based on the inclusion criteria presented in Table 6. To ensure privacy, we pseudonymized all participants, identified speakers by role (e.g., captain) or aircraft (e.g., UAL1), and excluded personally identifiable information (e.g., age) from the dataset. Given challenges in obtaining informed consent from individuals involved in incidents, we secured explicit permission from content creators/moderators of the public databases. As consuming conversations preceding aviation accidents may be traumatic, we prioritized already-transcribed conversations, ensured videos met YouTube community guidelines, provided close oversight to transcribers and analyzers, and offered the option of opting out of reading and transcribing distressing conversations. Our university research ethics board granted ethical approval.

**Table 5:** Data source characteristics.

Database	Description	Total number of incidents*	Years	Incident location	Data type	Incident type
National Transportation Safety Board**	USA government aviation database	176,172	1962–2024	USA	CVR and ATC transcripts	Fatal crashes Non-fatal crashes Near misses
Aviation Safety Network	Aviation incident database	~24,000	1919–2024	Worldwide	CVR and ATC transcripts	Fatal crashes Non-fatal crashes Near misses
VASAviation***	Aviation YouTube channel	1,122	2014–2024	Worldwide, though many from USA	ATC recordings	Fatal crashes Non-fatal crashes Near misses
Tailstrike.com	CVR transcript database	181	1965–2020	Worldwide	CVR transcripts	Fatal crashes Non-fatal crashes
Noort et al. (36)	Academic dataset	172	1962–2018	Worldwide	CVR and ATC transcripts	Fatal crashes Non-fatal crashes

ATC, Air Traffic Control radio transmissions; CVR, cockpit voice recorder; USA, United States of America.

*As of July 2024.

**To extract data from the National Transportation Safety Board, we used their search engine to download information on 23,778 incidents occurring between 1 January 2010 and June 28, 2024. We then scraped the database, downloading all PDFs containing the following keywords: transcript, CVR, cockpit, voice, recorder, ATC, conversation. Author 1 manually assessed the 1,297 resulting documents for eligibility.

***For conversations from VASAviation, tailstrike.com, and Noort et al. (36), we manually matched transcripts and incident reports using the Aviation Safety Network.

**Table 6:** Inclusion criteria.

Domain	Include	Exclude
Incident report	Has a final and/or preliminary incident report	No incident report
Conversation type	Flightdeck conversations preceding and including an aviation incident	Post-incident conversations (e.g., search and rescue efforts)
Data type*	CVR transcripts ATC transcripts ATC recordings	No or partial transcripts/recordings Conversation summaries Duplicates
Language	Transcripts/recordings and incident reports in English or professionally translated into English	All other languages
Aircraft type	Airplane	Helicopter Hot air balloon
Flight type	Commercial Personal Business/executive Sightseeing Sports team Military Cargo Positioning	Presidential Student/instructional Test Suicide
Incident type/cause	Mid-air collisions Ground collisions Runway incursions Runway excursions Controlled and uncontrolled flights into terrain/water Engine failures/fires Landing gear failures Missile launches Unwell pilots Near collisions	Hijackings/terrorism Unwell ATCs Unwell passengers Bomb threats Bird strikes

ATC, Air Traffic Control; CVR, cockpit voice recorder.

*If an incident had both CVR and ATC transcripts, we used the CVR transcript because it contained more conversational information.

Author 1 assessed cases based on the inclusion criteria presented in Table 6, and four psychology master’s-level research assistants completed transcriptions in Microsoft Excel. Since safety listening behaviors respond to safety voice acts, the research assistants identified relevant transcript sections by classifying instances of safety voice and the point of the incident. They transcribed a minimum of five lines of conversation before and after these markers for context, or from the start to finish of the transcript if necessary. We chose this method because certain transcripts or recordings contained many irrelevant conversations (e.g., the incident was at the landing stage, but the full transcript of conversations from take-off and during the long-haul flight were included).

We operationalized safety voice as the act of raising concerns about perceived hazards (5) with it being binary (i.e., utterances were either safety voice or not). Specifically, safety voice was considered present if a concerned team member raised a potential hazard or dangerous situation. We limited safety voice acts to those relevant to the incident’s cause, as identified *post hoc* in the incident report (e.g., fire, weather, navigation error, and air traffic control clearance). We did not consider standard communication practices (e.g., ATCs clearing aircraft for take-off) as safety voice unless there was a concern raised. Research assistants identified incidents’ outcomes in the transcripts using sounds (e.g., ‘[sound of impact]’), dialogs (e.g., another aircraft announcing to the ATC that an airplane crashed), aircraft being sufficiently distanced from each other (i.e., avoiding a collision), or the transcript’s end.

To ensure consistency, Author 1 and all research assistants indicated safety voice and the incident in the same five transcripts, and Author 1 provided feedback in the infrequent case when disagreements arose. Author 1 and the research assistants met biweekly for training, updates, and feedback. Author 1 quality-checked all transcripts and verified edge cases.

### 2.3 Taxonomy development

We analyzed 45 flightdeck conversation transcripts (6,009 lines; 49,526 words) preceding incidents from 1962 to 2023, sampling 15 each with outcomes of fatalities, aircraft damage without fatalities, and no damage or fatalities. Adopting a pragmatist approach (51), our analysis moved between deductive, inductive, and abductive frames. Using directed content analysis (52), we applied the deductive coding framework to identify safety listening behaviors, operationalized as observable responses to safety voice (6). We then inductively grouped and labeled similar behaviors, organizing clusters and identifying edge cases (53). Specifically, we examined instances of listening in transcripts and accident reports, comparing incidents to identify common, divergent, and unexpected behaviors beyond the deductive coding framework. Abductive theorizing (54) generated explanations for discrepancies between the deductive coding framework and the transcripts by confirming anomalies, proposing hunches, and testing them within the data. These steps produced version one of EARS, treating voice and listening acts as nominal variables.

We found gaps between the deductive coding framework and the data. The deductive framework crudely classified safety listening as (in)consistent or no responses to voiced requests; inductive coding indicated instances of ‘surface engagement’, where listeners verbally agreed with requests, yet their actions failed to align with their words (e.g., Table 1, Example 1). Thus, surface engagement is verbally consistent yet behaviorally inconsistent with voiced requests. We also found that disagreement—often conceptualized as poor listening [e.g., (36)]—was effective when voiced requests were incorrect or infeasible (e.g., Table 1, Example 3).

**Figure 2:**
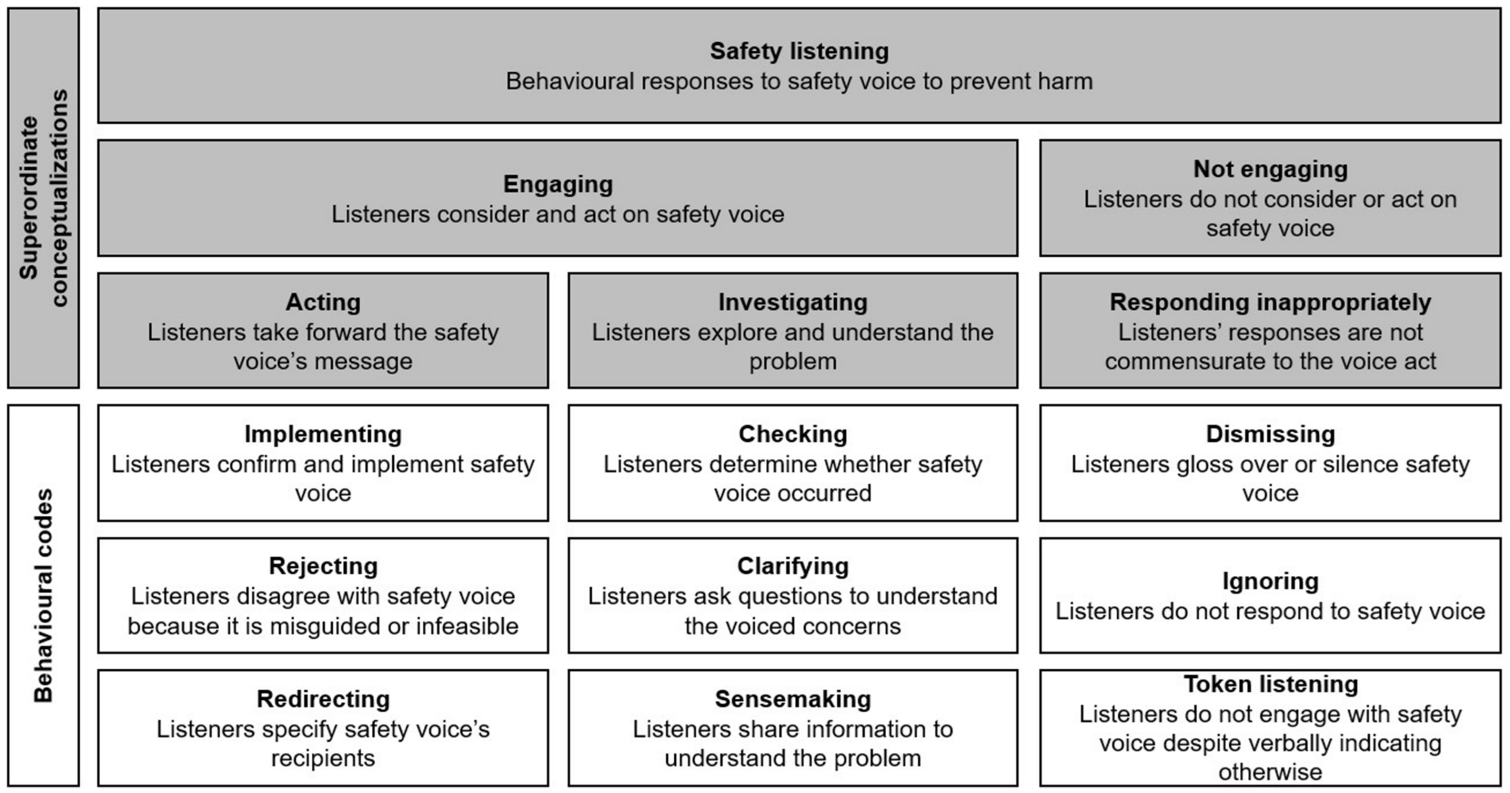
Initial safety listening behavior framework. Adapted from “Safety listening taxonomy.” Pandolfo, Reader, and Gillespie, under CC BY 4.0 (78).

From these findings, we developed a taxonomy that incorporates our insights into how listeners can engage in surface engagement and how effective listening may involve disagreeing with misguided concerns. This taxonomy distinguishes engagement and non-engagement with safety concerns (Figure 2). Engagement included ‘acting’ (implementing voice, declining misguided concerns, redirecting to appropriate parties) and ‘investigating’ (checking if someone spoke up, clarifying concerns, and sensemaking). Non-engagement comprised surface engagement, dismissing complaints, and ignoring concerns.

Sometimes individuals initiated additional voice acts in response to non-engagement (e.g., being dismissed). Voicers sometimes escalated their concerns with more urgency (‘escalating safety voice’; see Air China 428 in Table 2), or third parties amplified concerns by reiterating them more directly. For example, Air Canada 759’s cockpit—which had mistakenly aligned to land on a taxiway containing four aircraft—voiced that they saw ‘*some lights on the runway*’. The ATC assured them that ‘*There is no one on 28R [the runway] but you*’, failing to realize the problem. United 1 amplified Air Canada 759’s original concern, saying, ‘*Where’s this guy going? He’s on the taxiway!*’, prompting the ATC to request Air Canada 759 to abort their landing. We therefore added additional voice acts as behaviors following non-engagement with concerns. Pandolfo et al. (78) provide more detail about this analysis.

### 2.4 Taxonomy iteration

To refine EARS, we undertook an iterative process consisting of applying coding framework prototypes to unseen transcripts, troubleshooting, and adjusting measurement protocols (55). Each iteration combined quantitative (interrater reliability) and qualitative (coder feedback) evaluation of the tool’s performance, aligning with recommendations to use interrater reliability to uncover, investigate, and address areas of dissensus in qualitative coding (56).

We trained three master’s-level research assistants who were involved in transcription to apply EARS version one to flightdeck transcripts. Four coders (i.e., Author 1 and the research assistants) then independently applied EARS to batches of 10 transcripts, starting with incidents occurring in 2024 and working backward. After each batch, we solicited coder feedback—including challenging transcripts and edge cases—and calculated interrater reliability using Python. We measured interrater reliability using Krippendorff’s alpha and Gwet’s ACT1 because they account for zero values and multiple coders, respectively (57). We interpreted the scores as follows: 0.01–0.20 = poor/slight agreement; 0.21–0.40 = fair agreement; 0.41–0.60 = moderate agreement; 0.61–0.80 = substantial agreement; and 0.81–1.00 = excellent agreement (58). Integrating feedback, we revised EARS and repeated the process until saturation [i.e., minimal comments and revisions; (59)], resulting in the final version.

**Figure 3:**
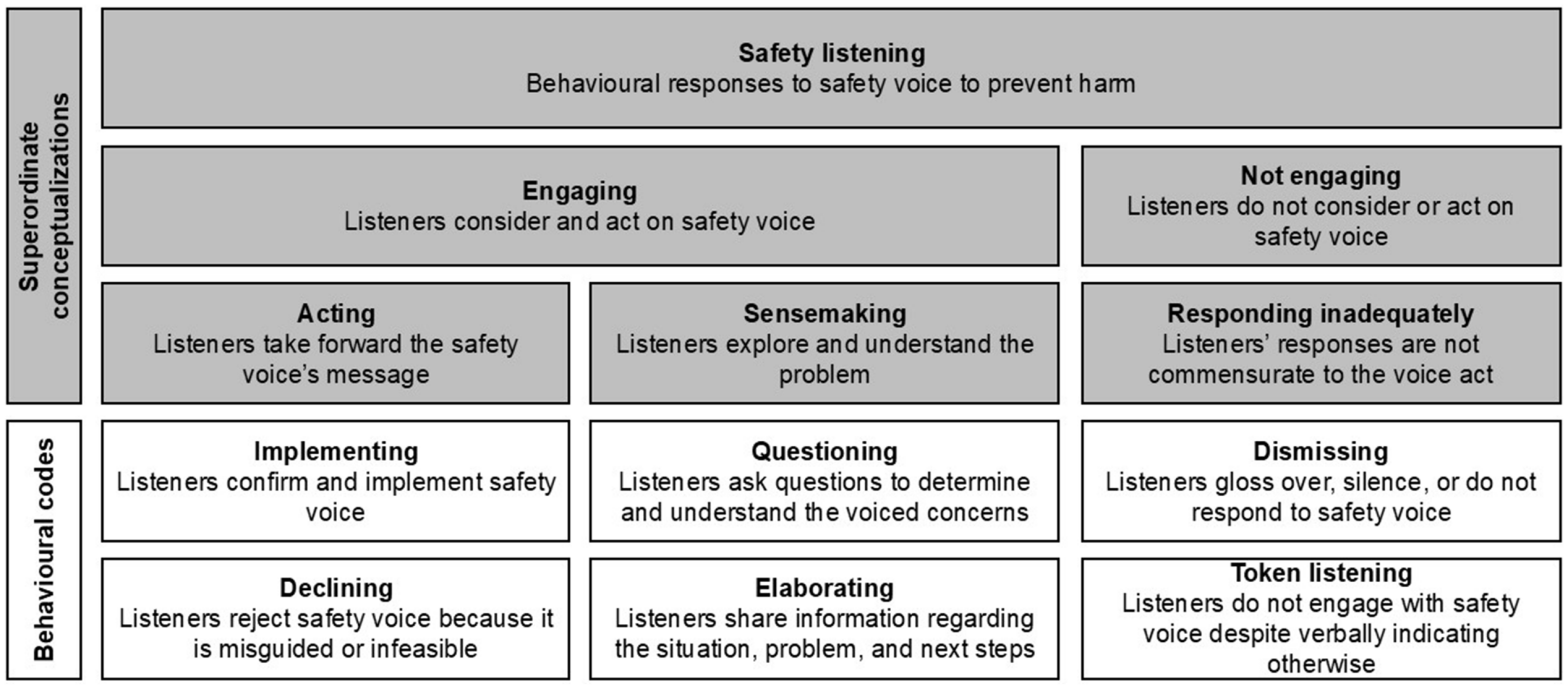
Final safety listening behavior framework. Reproduced from “Safety listening taxonomy.” Pandolfo, Reader, and Gillespie, under CC BY 4.0 (78).

EARS was finalized after four transcript batches (*n* = 40 transcripts, 2,244 lines, 19,433 words, from 2021 to 2024). Recognizing that some codes overlapped, we reduced the nine safety listening types to six (Figure 3). Specifically, we

(1) Recategorized ‘redirecting’ under ‘implementing’ because this behavior can be considered a form of implementation (i.e., understanding that the message was meant for a specific recipient and directing it to them);
(2) Combined ‘checking’ and ‘clarifying’ into ‘questioning’ because these behaviors involve asking whether someone spoke up and requesting more information about concerns;
(3) Merged ‘dismissing’ and ‘ignoring’ under ‘dismissing’ because these behaviors both can result in silencing voicers;
(4) Renamed ‘sensemaking’ as ‘elaborating’ because these behaviors served to build on the team’s understanding of the unfolding situation;
(5) Changed the category of ‘investigating’ to ‘sensemaking’ to better encompass the codes of ‘questioning’ and ‘elaborating’;
(6) Renamed ‘surface engagement’ as ‘token listening’; and
(7) Renamed ‘rejecting’ as ‘declining’.

We experimented with coding token listening separately (i.e., not as a type of listening) because identifying this listening form required an understanding of additional conversation turns or the accident itself. Separating token listening instead of classifying it as a listening type did not make a difference, and we kept it as a type of non-engaged listening.

We clarified that coders do not need to code every line between safety voice and the incident because some utterances following safety voice were irrelevant (e.g., the ATC giving routine updates). Likewise, some utterances could be classified under multiple codes, and we allowed coders to do so, but stressed that, where possible, one code should be used.

The final EARS coding framework with examples is presented in Table 7, and the full manual is freely available to download (Supplementary material 1). The last batch of transcripts analyzed in this phase had an overall Krippendorff’s alpha of 0.73 and a Gwet’s ACT1 of 0.80.

**Table 7:** Summary of the Ecological Assessment of Responses to Speaking-up coding framework.

Superordinate conceptualization	Code	Coding*	Example	Interpretation
Safety voice		A concerned team member raised a potentially dangerous situation or hazard	Atlas Air GTI95: ‘MAYDAY MAYDAY, Giant 095 heavy, engine fire. Request vectors back to the airport’.	The aircraft crew realized that their engine was on fire and asked to return to the airport.
Safety listening	Implementing	Agreeing and acting on the concern. Includes giving new or updated instructions, redirecting voice to specific recipients, confirming courses of action or plans, giving permission for a new or changed course of action, acknowledgments, and answering questions by changing the course of action	WestJet 2425: Stop Sunwing’. ATC: ‘Tractor 540 stop there’.	A tractor was pushing a Sunwing airplane toward a collision with WestJet 2425. The WestJet crew instructed Sunwing plane to stop, and the ATC specified that a tractor should push the plane.
	Declining	Understanding and disagreeing with voiced requests or the voicer’s understanding of the situation. Also, being unable to act on voice	LaMia 2933: ‘Vectors to the runway!’ ATC: ‘We lost radar signal. I cannot see you’.	LaMia 2933 was landing with electric and fuel failures, and the cockpit requested that the ATC give them vectors to the airport. The ATC declined, saying they could not give vectors without a radar signal.
	Questioning	Determining whether colleagues had spoken up, asking for repetitions or clearer communications, and asking for more information. Includes asking for repetitions, saying that they are having difficulty hearing voicers, asking if they raised a concern, asking questions to get more information about the problem, giving alternative solutions or next steps, and asking if aircraft can hear them	Transair 810: ‘We have lost number one engine and we are coming straight to the airport we are going to need the fire department there is a chance we are going to lose the other engine too. It’s running very hot and ah… speed is ah… we are pretty low on speed. It does not look real good up here, you might want to let the coast guard know as well. And we do not have any hazmat and ah… fuel is about 2 h of fuel’. ATC: ‘And Rhoades Express 810, how many people are on board?’	Transair 810 told the ATC that they had lost their engine and that they were returning to the airport. The ATC sought further details (i.e., souls on board).
	Elaborating	Verbally sharing updates about the situation, problem, or next steps. Includes expanding on the problem, answering questions asked without a change of action, sharing possible explanations of the problem, sharing information, sharing ideas for next steps, getting more information, and giving facts	HopAJet 823: HopAJet 823, lost both engines. Emergency. Making an emergency landing. ATC: Was that an emergency? Cleared to land runway 23. Is that HopAJet 823? HopAJet 823: Yeah, we are cleared to land but we are not gonna make the runway. We’ve lost both engines.	HopAJet 823 voiced that it lost its engines and was making an emergency landing. The ATC engaged in questioning, to which the HopAJet cockpit elaborated that they would not make the runway as they lost both engines.
	Dismissing	Includes irrelevant responses to speaking up by the intended listener, problems with hearing voice, defensiveness (i.e., avoiding, delegitimizing, limiting), retaliation, rudeness, failing to consider the voiced request, and silencing voicers	First Officer: ‘Sir, should not I switch on the radar?’ Pilot in Command: ‘That fucker Lamia, she made me… look at my fucking eyes… weeping… crying…’	In US-Bangla Airlines 211, the Pilot in Command had a breakdown mid-flight about a rumor that he and a colleague of him had sex in the cockpit. The First Officer was trying to fly the aircraft; however, the Pilot in Command was not engaging with her concerns.
	Token listening	Listeners verbally acknowledge safety voice, but their actions indicate a lack of thorough consideration of the voiced content. This is evidenced by inconsistencies between listeners’ verbal responses and their actions	ATC: ‘Additional traffic north shore, it’s a Metroliner for the parallel’. N416DJ: ‘I have traffic in sight, cleared to land 17R, 6DJ’.	The ATC warned N416DJ about another aircraft (i.e., Key Lime 970). N416DJ said they saw the traffic; however, they collided mid-air with Key Lime 970 shortly afterwards.
Additional safety voice act	Escalating safety voice	After safety voice and safety listening—the original voicer raises the same safety concern, often in a clearer and more direct way	ATC: ‘climb immediately, maintain 4,000’. N7022G: ‘4,000’, climbing immediately’. ATC: ‘Okay, it looks like you are descending, sir. I need to make sure you are climbing, not descending’.	The ATC told N7022G to climb, and the pilot verbally confirmed. However, the pilot descended the plane, highlighting a disconnect [token listening]. The investigation revealed that the pilot had lost spatial orientation. The ATC engaged in escalating voice, emphasizing that the aircraft must climb.
	Amplifying safety voice	After safety voice and safety listening—a third party raises the same safety concern, often in a clearer and more direct way	ACA759: ‘And Tower, just wanna confirm—it’s Air Canada 759, we see some lights on the runway there. Please, confirm we are cleared to land?’ ATC: ‘Air Canada 759, confirm. Cleared to land runway 28R. There is no one on 28R but you’. ACA759: ‘Okay, Air Canada 759’. UAL1: ‘Where’s this guy going? He’s on the taxiway!’	United 1, an aircraft on the taxiway, noticed that Air Canada 759 was aligned with the taxiway rather than the runway. Amplifying voice reinforced the Air Canada cockpit’s muted concern about seeing lights, helping to avoid a multi-aircraft collision.
Point of incident		The moment at which the incident occurred or was averted, identified through sounds, dialogs, aircraft positioning, or the transcript’s end	Watch supervisor: ‘Okay I have an emergency actually it’s an aircraft incident the aircraft just went down’.	In Sniper 1, the aircraft lost control, and a controlled flight into terrain occurred.

### 2.5 Taxonomy testing

The final version of EARS was tested in three ways. First, we applied EARS to an unseen dataset, using the same three research assistant coders as in the taxonomy iteration. The coders applied the final version to 110 unseen flightdeck transcripts. These transcripts began from the point where taxonomy iteration stopped, and Author 1 and the coders met approximately biweekly to discuss feedback and edge cases. Interrater reliability was calculated as described above. Included incidents occurred between 2016 and 2021, and transcripts consisted of 10,560 lines and 100,062 words. The interrater reliability scores for all codes in these transcripts were a Krippendorff’s alpha of 0.77 and a Gwet’s ACT1 of 0.87. Table 8 provides a code-by-code breakdown.

**Table 8:** Reliability of three raters coding 110 flightdeck transcripts.

Superordinate conceptualizations	Code	Absolute agreement (%)	Krippendorff’s alpha	Krippendorff’s alpha 95% CI	Gwet’s ACT1	Gwet’s ACT1 95% CI	Rater 1 code frequency	Rater 2 code frequency	Rater 3 code frequency
Safety voice		100	0.96	0.94–0.98	1	1.00–1.00	112	111	111
Safety listening	Implementing	91	0.63	0.61–0.65	0.88	0.88–0.89	1,611	1,598	1,596
	Declining	99	0.24	0.17–0.30	0.99	0.99–1.00	51	2	72
	Questioning	98	0.83	0.81–0.85	0.98	0.97–0.98	703	797	736
	Elaborating	92	0.59	0.57–0.61	0.89	0.89–0.90	1,309	1,446	1,231
	Dismissing	100	0.19	−0.01–0.40	1	1.00–1.00	4	6	16
	Token listening	100	0.21	−0.01–0.42	1	1.00–1.00	14	4	6
Additional safety voice acts	Escalating	100	0.53	0.39–0.66	1	1.00–1.00	17	27	30
	Amplifying	100	0.00	0.00–0.00	1	1.00–1.00	2	2	5
Incident		100	0.98	0.97–1.00	1	1.00–1.00	111	111	112

Second, a psychology master’s-level coder—who was uninvolved in transcription and taxonomy iteration—independently coded 50 transcripts using the final tool. These transcripts were a subset of the 110 and all occurred in 2019 and 2020 (*n* = 30 and 20, respectively). Before coding, the coder received training which included explaining the final coding framework with examples and coding three transcripts with Author 1. Midway through coding, the coder and Author 1 met to discuss edge cases and coded an additional transcript together. Interrater reliability was calculated as described above. The data consisted of 4,824 lines and 43,936 words. This analysis’ interrater reliability scores for all codes were a Krippendorff’s alpha of 0.76 and a Gwet’s ACT1 of 0.87.

Third, we identified transcript lines where all coders disagreed in the previous two analyses. Using inductive thematic analysis (60), we formed themes at the semantic level summarizing content where the EARS tool resulted in discrepant coding. We found that all coders disagreed 169 times in the first analysis (larger unseen dataset) and 20 times in the second (new coder). Similar to the findings by MacPhail et al. (61), we noted that in most instances where coders had used different codes, the codes were from the same thematic group (e.g., engagement with voice). As illustrated in Table 9, coders had difficulty when communications were ambiguous (e.g., unintelligible utterances), transcript lines contained aspects which conformed to multiple codes (e.g., declining suggestions and asking questions), and/or it was unclear whether listening acts occurred (e.g., confirming one’s attention).

**Table 9:** Types of instances resulting in discrepant coding.

Theme	Sub-theme	Description	Example	Coder 1	Coder 2	Coder 3
Ambiguous communications	Unintelligible communications	Parts of what was said cannot be determined	Air Astana 1388: ‘Air Astana 1388 [unintelligible]’	Implementing	None	Elaborating
	Sounds	Utterances like um, ah, uh, and erm.	Eastern Air Lines 3452: ‘ahhhh…’.	Elaborating	Implementing	None
	Equivocal communications	The message can be interpreted in multiple ways	American 383: ‘speed’.	Implementing	Elaborating	Escalating
Multiple codes		One transcript line which includes multiple potential codes	N22AM: ‘two alpha mike no ahh… I am in the middle of the clouds what is my current heading’	Elaborating	Questioning	Declining
Ambiguous listening acts	Requesting attention	An individual asking another for attention	Atlas Air Cargo 3591: ‘@Capt. [Spoken in elevated voice.]’	Questioning	Elaborating	None
	Confirming attention	An individual confirming their attention	Orlando Tower: ‘N1958—correction—N1958R, Orlando Executive Tower’	Elaborating	Implementing	None

## 3 Discussion

In this article, we developed, iterated, and tested the EARS tool—a reliable assessment of safety listening behaviors in naturalistic and high-risk conversations. The research aimed to understand and improve responses to safety voice, thereby enhancing safety in risky organizations. Raising and responding to safety concerns are crucial for averting organizational disasters (5, 6), yet to date, there has been no reliable tool for classifying naturalistic behaviors following speaking up. Such a tool is necessary for scholars to empirically examine safety listening behaviors, addressing calls for balancing the use of self-report and behavioral measures (6, 28).

Measuring safety voice and safety listening has conceptual and operational difficulties. Voice and listening are typically measured using self-reports despite both being observable behaviors, likely due to these concepts’ elusiveness and difficulties capturing them naturalistically (5, 6). Moreover, safety listening requires the occurrence of safety voice; there is no listening without speaking up. Thus, developing a safety listening behavioral framework requires a reliable and valid voice typology to identify safety voice acts. Here, we coded safety voice consistent with Noort et al. (36) which conceptualized safety voice behaviors as binary (i.e., voice or silence).

The EARS tool comprises six forms of safety listening behavior and two additional safety voice acts. Its components include response behaviors identified in previous empirical studies [e.g., content is implemented; (12)]; however, rather than positioning effective listening as agreeing with voicers, EARS posits that effective listening engages with voice. As such, it goes against the prevailing assumption in the literature that listening is attitudinal (i.e., individuals did not listen because they did not want to), moving toward a conceptualization of listening as skill based, with the effectiveness of behavior hinging on the context and situation. The engagement behaviors captured by EARS include implementing suggestions, declining misinformed safety voice, questioning voicers, and elaborating on their understanding of the situation. Conversely, non-engagement included dismissing or ignoring voicers, and token listening—where listeners’ verbal responses are inconsistent with their actions.

We also identified two additional safety voice acts that occurred after poor safety listening: escalating and amplifying voice. These additional voice acts illustrate processes through which poor listening may be rectified via clearer and more direct subsequent conversation turns and third parties amplifying initial concerns.

### 3.1 Theoretical and practical implications

Creating the EARS tool will improve safety listening’s conceptual clarity (40), allow for comparable empirical findings, and reliably analyze safety communication in hitherto neglected large public behavioral datasets [e.g., Flint water crisis emails; see Pandolfo et al. (6) for other possible datasets]. By analyzing pre-incident conversations, we aim to glean insights into their influence on the likelihood of accidents. Ultimately, this tool can be used in organizations to give feedback, analyze incidents, and score simulations. It can also be integrated into various training programs (e.g., crew resource management) and inform policy decisions, thereby contributing to safety standards’ continuous improvement.

EARS can be used to develop interventions and assess listening behaviors pre- and post-intervention (e.g., measuring status quo safety listening in organizations to establish a baseline against which to evaluate this behavior during and after interventions). Like non-technical skills (62), crew resource management (63), and workplace listening (64) programs, listening is a trainable skill. Accordingly, training effective safety listening behaviors may reduce harm in high-risk organizations like aviation. For instance, training programs may teach strategies for clarifying hinted-at concerns, disagreeing with misdirected voice, and addressing multiple voicers delivering conflicting information. The program should incorporate real conversation recordings to help participants assess how others in their roles communicate effectively and how they could concretely improve (65).

Assessing intervention effectiveness may be aided by developing a large language model (LLM) text classifier to identify safety listening behaviors. At present, EARS is a reliable manual coding system. LLM text classifiers can be used to quickly code datasets consisting of thousands of conversation transcripts. Developing classifiers could be done using prompt engineering with LLMs like GPT-4. Researchers should iterate on prompts (e.g., ‘do any lines of transcript engage with a safety concern?’) to instruct the LLM on how to classify the textual data and test its accuracy against manually coded qualitative data. An automated system could run in real-time (i.e., coding listening behaviors during high-risk interactions). The LLM’s generalizability and misclassifications should be assessed to determine the prompt’s validity in reproducing the manual coding framework.

Although EARS was developed in aviation, there are promising opportunities for its application in other contexts. These include assessing co-located teams in other high-reliability organizations, lone workers, and responses to promotive safety voice (Table 10). Likewise, it could be adapted into a standard and valid Likert-type survey to create a scalable, low-cost measurement of individual and team listening behaviors. For instance, as safety voice climate scales exist [e.g., (66)], a counterpart ‘safety listening climate’ scale could be created. While this survey would risk biases (e.g., self-report) and require cross-industry validation, it would enable benchmarking, soliciting multiple perspectives (e.g., self, peer, supervisor), and linking these perceptions to coded behavior.

**Table 10:** Suggested extensions for the Ecological Assessment of Responses to Speaking-up tool.

Extension	Examples	Rationale	Considerations	Suggestions
Teams in high-reliability organizations	Railway maintenance Surgery Chemical plants	Assesses the generalizability of listening behaviors to other co-located teams Facilitates cross-industry benchmarking	Sector-specific jargon and communication styles (e.g., medical terms) Ethical, access, and privacy barriers to data collection (e.g., patient dignity) Variable outcome metrics (e.g., environmental damage, patient experience)	Begin with *in situ* observations or archival conversations [see Pandolfo et al. (6) for possible datasets], applying EARS and noting gaps in the coding scheme Adapt EARS to incorporate nuances and domain-specific behaviors Pilot and validate the updated tool
Lone workers	Field technicians Truck drivers Offshore inspectors	Expands beyond co-located team-based settings to capture remote, technology-mediated listening Supports trust and safety in isolated roles	Sparse and asynchronous communications may be difficult to capture	Consider text messages or emails, identifying gaps, adapting EARS, and piloting it Note that listening may occur over time, with listeners becoming future voicers (75)
Promotive and prosocial safety voice (17, 76)	Suggestions for future safety-related improvements	Shifts focus from avoiding harm to continuous organizational improvement	Less urgent than the preventive voice Outcomes may be long-term, subtler, and more challenging to link with conversations than in aviation	Consider that codes like ‘elaborating’ may be irrelevant because sensemaking may not occur Collect longitudinal data to determine if suggestions are implemented [like the methodology used in Satterstrom et al. (50)]

### 3.2 Limitations and future directions

This research has limitations. First, raters had difficulty coding ambiguous transcript lines, specifically those with unclear utterances (e.g., unintelligible parts), content which encompassed multiple codes, and situations where speakers were requesting or confirming attention. In particular, the codes of declining, dismissing, and token listening had notable between-rater frequency differences, possibly due to coinciding with other codes in the same utterance. These codes were also rare (i.e., less than 100 instances for each); raters may have developed conflicting ideas about their inclusion/exclusion because they did not have as much practice or feedback with them. These situations should be considered when applying EARS to other transcripts and datasets.

Second, despite our best efforts to include global transcripts, most (128/150) incidents occurred in American airspace. This skew is likely caused by three factors. First, the United States’ National Transportation Safety Board database had the largest number of publicly available transcripts and incident reports, and most of the VASAviation recordings occurred in America. Second, some countries (e.g., the UK) legally prohibit the public from recording ATC feeds, meaning that their airports have few publicly available recordings (41). Third, some governmental agencies (e.g., Transportation Safety Board of Canada) have policies that exclude flightdeck conversation transcripts from incident reports as they consider these data to be privileged. As flightdeck communication varies by national culture (67), future studies should investigate EARS’ cross-cultural generalizability.

Third, EARS was developed using transcripts of flightdeck conversations. We recognize that pilots, ATCs, and other aviation staff are trained in communication through initiatives like crew resource management. Consequently, individuals in our transcripts may have exhibited more engagement with concerns than those in other contexts. Flightdeck communications are urgent and time-sensitive, with crew members collaborating transparently as a team, while pilots face direct physical risks in the event of a crash. Accordingly, we found the most common codes were implementing, elaborating, and questioning. Conversely, an analysis of speaking-up and responses in the Mid Staffordshire hospital scandal (1,200 died from poor healthcare) found that the commonest response to speaking-up was inaction [i.e., acknowledging a concern but not correcting it; (14)]. EARS would classify the non-responses as non-engagement, specifically ‘dismissing’. With appropriate modifications, we believe that EARS can be generalized to code safety listening in written responses to complaints, *in situ* observations, and in non-aviation contexts (e.g., surgical teams). Following the suggestions presented in Table 10, future studies should investigate safety listening in other contexts to discern how these factors influence engagement with concerns.

Fourth, piloting an airplane is not entirely verbal; therefore, there may be actions which are not captured in the transcripts. For instance, a solo pilot may silently run through a checklist; however, this action would not be captured on the transcript unless the pilot announced this behavior to the ATC.

Last, EARS assesses safety listening behavior in naturalistic conversations. While we encourage safety listening researchers to assess behavior in naturalistic data, we recognize that this data type cannot assess attitudes, rationales, or felt emotions, which might influence outcomes. Self-report measures are necessary for assessing such intentions and psychological states. Like Barnes et al. (68), we recommend that future research triangulate behavioral and self-report data to develop deeper insights into the relationship between psychological states, communications, and incidents. One possibility within aviation would be for scholars to analyze aviation incidents in the National Transportation Safety Board database, for which there are (1) incident reports, (2) *post hoc* interview transcripts with relevant parties (e.g., pilots and ATC), and (3) CVR or ATC transcripts.

## 4 Conclusion

In this article, we developed and tested the Ecological Assessment of Responses to Speaking-up (EARS) tool. This assessment provides a reliable and theoretically grounded framework through which safety listening acts can be identified, learned from, and investigated in relation to speaking-up and incident outcomes. In contrast to the idea that effective listening agrees with voicers, EARS emphasizes the importance of engaging with safety voice. EARS identifies six safety listening behaviors: action (implementing and declining), sensemaking (questioning and elaborating), and non-engagement (dismissing and token listening), and two additional safety voice acts: escalating and amplifying safety voice. We demonstrate that this tool achieved substantial interrater reliability and allows for the assessment of safety listening behaviors in naturalistic, high-risk conversations. EARS can be used to analyze safety listening’s antecedents, its relationship with safety voice and outcomes, and pre- and post-intervention safety communications to improve organizational safety.

## Data Availability

The raw data supporting the conclusions of this article will be made available by the authors, without undue reservation.
