# Supplementary material for: The Ecological Assessment of Responses to Speaking-up tool—development and reliability testing of a method for coding safety listening behavior in naturalistic conversations
Source: Front Public Health. 2025 Oct 14;13:1652250. doi: 10.3389/fpubh.2025.1652250 (PMC12560795; doi:10.3389/fpubh.2025.1652250)
Supplement: Supplementary file 1 [file Table_1.docx]

**The Ecological Assessment of Responses to Speaking-up (‘EARS’) manual**

Alyssa M Pandolfo, Tom W Reader & Alex Gillespie

London School of Economics and Political Science

Department of Psychological and Behavioural Science

# INTRODUCTION

This codebook focuses on aviation team safety communications and situation awareness. The dataset comprises excerpts of transcripts of conversations preceding aviation incidents. The excerpts begin shortly before a team member engaged in safety voice and end shortly after incident outcomes (i.e., a crash or near miss). This document explains the variables to code.

Before transcription, research assistants identified the first instance of safety voice in the conversation. We defined safety voice as the act of raising concerns about perceived hazards (Noort et al., 2019) with it being binary (i.e., utterances were either safety voice or not safety voice). Specifically, safety voice was considered present if a concerned team member raised a potential hazard or dangerous situation. We limited safety voice acts to those relevant to the incident’s cause, as identified post-hoc in the incident report (e.g., fire, weather, navigation error, air traffic control clearance). We did not consider standard communication practices (e.g., ATCs clearing aircraft for take-off) as safety voice unless there was a concern raised. We included incidents without safety voice in our dataset; however, we did not transcribe or analyze them. Table 1 illustrates examples of safety voice in aviation transcripts.

Research assistants identified incidents’ outcomes in the transcripts. Outcomes were identified through sounds (e.g., “[sound of impact]”), dialogues (e.g., another aircraft announcing to the ATC that an aircraft crashed), aircraft being sufficiently distanced from each other (i.e., avoiding a collision), or the transcript’s end. Incidents were coded as ‘accident’ if there was a crash (i.e., at least one fatality and/or airplane damage) and ‘incident averted’ if there was a near miss (i.e., no fatalities and no airplane damage).

Research assistants transcribed a minimum of five conversation lines before the first act of safety voice and after the incident. The exception to this was if there were fewer than five transcript lines available pre-safety voice or post-incident.

# Table 1: Safety voice examples

|  | **Quote** | **Interpretation** |
| --- | --- | --- |
| 1 | “I've got a serious situation here about my pilot. He's incoherent, no idea how to fly the airplane but I'm maintaining 9,100'.” (Passenger, Caravan 333LD) | The passenger tells the ATC that the situation is no longer business as usual – the pilot is incapacitated, and they do not know how to fly the airplane. |
| 2 | “Postman 1491, MAYDAY MAYDAY MAYDAY, request level off at 6000'.” (Cockpit, DHL 1491) | The ‘maydays’ indicate that the situation is no longer business as usual (‘strong safety voice’) and that instead of continuing to climb after take-off, DHL 1491 wishes to level off. |
| 3 | “Transavia 5193, left turn immediately heading 180.” (ATC, KLM 1080/Transavia 5193) | The ATC tells Transavia 5193 to turn left to avoid collision with another aircraft. |
| 4 | “smells like wood burning. smell that?” (First Officer, UPS 1307) | The First Officer points out a strange smell and asks others if they also smell it. |
| 5 | “we're now (at take-off)” (First Officer, KLM 4805) | This message is ambiguous (‘muted safety voice’) – the First Officer intends to communicate that KLM 4805 is taking off without take-off clearance; however, the ATC and other aircraft interpret it as ‘we are at take-off position’. |
| 6 | “Where’s [Mount] Erebus in relation to us a[t] the moment?” (First Officer, Air New Zealand 901) | The First Officer hints their concern about hitting the mountain (muted safety voice). |
| 7 | “Japan air niner zero seven, descend and maintain flight level three five zero, begin descent due to traffic” (ATC, Japan Air Lines 907) | The ATC mistakenly tells Flight 907 to descend despite meaning to tell Flight 958 to descend (‘misinformed safety voice’). |
| 8 | “It must be the trim.” (Captain, United 9963) | The captain mistakenly believes the trim is incorrectly set when the true problem was the flap settings (‘misinformed safety voice’). |

# SAFETY LISTENING

Safety listening is a behavioural response to safety voice in organizational contexts to prevent harms (Pandolfo et al., 2024). We conceptualize safety listening in terms of behaviours engaging with safety voice acts, where listeners consider and take forward the voiced information. Engagement manifests through *action* and *sensemaking*. Conversely, poor safety listening is characterized by non-engagement, where listeners disregard safety voice, leading to no safety improvements.

Safety listening is in the next relevant conversation turn after safety voice. This typically is in the following conversation turn after safety voice; however, occasionally other irrelevant utterances (e.g., ATC giving another cockpit directions) may be in the following turn. For example, a first officer may privately ask the captain if he smells a strange odour (safety voice), and before the captain can respond, the ATC over the radio clears another aircraft for takeoff. The takeoff clearance is not safety listening because the ATC (1) did not hear the voice and (2) was not the voice’s intended recipient. The captain’s response (or lack thereof) would constitute safety listening.

Please indicate which type of safety listening is present in the ‘event’ column in the Excel spreadsheet. If an utterance is not safety listening, please leave the cell blank. If you believe that two codes fit the transcript line, you may use both; however, where possible, please use one. Please note that voicers and listeners may switch roles in the transcripts. Voicers may engage in listening behaviours like elaborating while initial listeners may amplify initial safety voice acts.

## Engaging with safety voice

### Acting on safety voice

The first type of engagement was action, where listeners indicated that they heard the voice act and would maintain safety.

#### Implementing

Implementation behaviours involved listeners agreeing with and acting on the voicer’s concern. Listeners often verbally confirmed voice requests and acted accordingly (Table 2, examples 1-2) or gave voicers permission to change their course of action (Table 2, example 3). Sometimes, listeners would act on safety voice without verbal acknowledgement (Table 2, examples 4-5). Redirecting voice (i.e., listeners direct voiced information to specific recipients) is included here.

Implementing is agreement and action. It is a change in the course of action. It is different from elaborating, as elaborating is purely verbal, reporting on what is happening and sharing information about the problem/situation/next steps.

##### Inclusions

Giving new or updated instructions (e.g., new radio frequencies, telephone numbers, new vectors, cancelling approach clearance, ‘climb to 2,000’), redirecting voice to specific recipients (e.g., specifying that a voice act was meant for the ATC), confirming courses of action or plans (e.g., ‘going around’, ‘stopping take-off’, ‘climbing’), giving permission for a new course of action or changed course of action (e.g., ‘permission given for standard missed approach’, ‘new altitude of 4,000’ granted’), acknowledgments (e.g., ‘roger’, ‘okay’, ‘copy that’, ‘I agree’), answering questions by changing the course of action (e.g., responding ‘yes I’m turning left now’ to ‘are you turning left?’).

##### Exclusions

Asking questions (e.g., ‘is the smell coming from the back?’; this would be questioning), giving alternative solutions (e.g., ‘do you want to go to LaGuardia Airport or Newark Airport?’; this would be questioning), sharing updates about the situation (e.g., ‘there is precipitation near the airport’; this would be elaborating), electronic voices (this would be elaborating), answering questions without acting (e.g., ‘yes there is precipitation at the airport’; this would be elaborating), expanding on safety concerns (e.g., ‘we have lost another engine’; this would be elaborating), disagreeing with proposed actions (this would be declining).

# Table 2: Safety listening as implementing safety voice

|  | **Transcript excerpt** | **Interpretation** |
| --- | --- | --- |
| 1 | ATC: “Velocity 669, stop! Stop! You seem to be taking off on [runway] 30 there.”  Virgin Australia 669: “669 stopping.” | The aircraft began taking off from the wrong runway. The ATC intervened and the crew halted the take-off. |
| 2 | ATC: “Shit! Delta 1943, cancel takeoff clearance.”  Delta 1943: “Rejecting. Alright, oof, Delta 1943.” | Delta 1943 was taking off when another aircraft was crossing the same runway. The ATC intervened and the crew confirmed the stop and acknowledged the near collision. |
| 3 | American Airlines 2172: “American 2172 going around.”  ATC: “roger. American 2172, turn right heading 270.” | American Airlines 2172 was cleared to land on the same runway as another aircraft taking-off. The crew noticed this and requested a go-around. The ATC confirmed and gave instructions for a missed approach. |
| 4 | ATC: “Delta 873, cancel takeoff clearance.”  [Delta 873 did not verbally respond but halts the takeoff] | The ATC noticed that Delta 873 was on a collision course with another aircraft on the runway and cancelled their takeoff clearance. Although Delta 873 did not verbally confirm, they demonstrated their understanding by halting the takeoff. |
| 5 | ATC: “Foxtrot Victor, hold position here!”  [N990FV did not verbally respond but stopped the aircraft] | The ATC observed N990FV crossing in front of another aircraft during takeoff and told them to stop. Although N990FV did not verbally confirm, they demonstrated their understanding by halting the takeoff. |
| 6 | WestJet 2425: “Stop Sunwing.”  ATC: “Tractor 540 stop there.” | A tractor was pushing a Sunwing airplane towards a collision with WestJet 2425. The WestJet crew said to stop the Sunwing plane, and the ATC specified the tractor pushing the plane, requesting them to stop. |
| 7 | Unidentified aircraft: “Tower, there is a hawk lying between the parallels.”  ATC: “Attention all aircraft, use caution for hawk reported between the parallels.” | The aircraft raised the concern that there was a hawk between the parallels to the ATC, who redirected it to all aircraft on the open channel. |
| 8 | United Airlines 1: “Where’s this guy going? He’s on the taxiway!”  ATC: “Air Canada, go around.” | Air Canada 759 accidentally lined up for landing on taxiway C (containing four aircraft) instead of runway 28R. United Airlines 1 communicated this error on the open channel and the ATC told Air Canada 759 to abort landing. |

#### Declining

The second form of action is where listeners reject safety voice when they (1) believe voicers were wrong and/or (2) could not act on the information. Phrases indicating rejection include “no”, “can’t”, “unable”, and “negative” (Table 3, examples 1-4).

##### Inclusions

Understanding and disagreeing with voiced requests or the voicer’s understanding of the situation, being unable to act on voice

##### Exclusions

Rejections for the sake of rejecting or failing to consider the voiced request (this would be dismissing)

# Table 3: Safety listening as declining safety voice

|  | **Transcript excerpt** | **Interpretation** |
| --- | --- | --- |
| 1 | Captain: “It must be the trim.”  First Officer: “No, it’s in the green band now. It can’t be the trim.” | A warning horn signalled an incorrect take-off setting (i.e., the flaps). The captain wrongly suggested the problem was the trim and the first officer corrected him. |
| 2 | ATC: “Cactus fifteen twenty-nine, if we can get it for you do you want to try to land runway one three?”  US Airways 1549: “we're unable. we may end up in the Hudson [River].” | US Airways Flight 1549 struck birds shortly after take-off and damaged the engines. The ATC suggested runway options while the captain disagreed and said they will land in the Hudson River – which they eventually did. |
| 3 | LaMia 2933: “Vectors to the runway!”  ATC: “we lost radar signal. I can’t see you.” | LaMia 2933 was landing with electric and fuel failures, and the cockpit requested that the ATC give them vectors to the airport. The ATC declined, saying they could not give vectors without radar signal. |
| 4 | ATC: “Southwest 708, roger, you can turn right when able.”  Southwest 708: “Negative.” | Southwest 708 was taking off when another aircraft was landing on the same runway. The ATC told the crew to turn right and the crew declined as they were unable to do so. |

### Sensemaking safety voice

The first type of engagement with safety voice was action; the second type is sensemaking. Sensemaking involves teams recognizing that a voice act has occurred and then attempting to understand its reasons and content. Sensemaking has two types: questioning and elaborating.

For joint sensemaking/problem-solving, you can code these as ‘elaborating/questioning’ if the utterance covers both sensemaking types. For example, in N2104J, the ATC said “it'd be less [or easier] terrain as well if you decide to go to Charlottesville. Like I said it's about 20 miles northeast of you. You wanna continue to Eagle Nest or Charlottesville?” – the first part of the utterance is elaborating, and the second part is questioning (i.e., where they ask which airport they’d like to go to). You can code these as ‘elaborating/questioning’ because this speech act does both.

#### Questioning

Questioning encompasses teams determining whether colleagues had spoken up, asking for repetitions or clearer communications, and asking for more information. Table 4 has examples.

##### Inclusions

Asking for repetitions (e.g., ‘I missed that, what altitude do we climb to?’), saying that they are having difficulty hearing voicers (e.g., ‘all I’m hearing is static’), asking if they raised a concern, asking questions to get more information about the problem (e.g., ‘how many souls on board?’, ‘how much fuel in pounds do you have left?’), giving alternative solutions or next steps (e.g., ‘Do you want to go to LaGuardia Airport or Newark Airport?’), asking if voicers can hear them (e.g., ‘Castle 921 are you there?’).

##### Exclusions

Answering questions, giving facts, giving updates about the situation/problem/next steps (these are all in ‘Elaborating’).

# Table 4: Safety listening as questioning

|  | **Transcript excerpt** | **Interpretation** |
| --- | --- | --- |
| 1 | Southwest 1380: [static noises]  ATC: “Southwest thirteen eighty if you’re trying to get me all I hear is static.” | Southwest 1380 experienced an engine fire and attempted to contact ATC to declare an emergency; however, the ATC could not hear them well. |
| 2 | Flight engineer: “Is he not clear then?”  Captain: “What do you say?” | The flight engineer of KLM 4805 asked whether the other aircraft had cleared the runway that they were taking-off from. The captain asked for repetition and clarification. |
| 3 | First officer: “Lightning coming out of that [cloud].”  Captain: “What?” | Delta 191’s first officer pointed out lightning and the captain asked for repetition and clarification. |
| 4 | Alaska Airlines 322: “aircraft just passed off our right, and it’s rolling.”  ATC: “What’s the aircraft on Runway 16C?” | In Horizon Air (hijacked airplane), Alaska Airlines 322 raised the concern about an unidentified aircraft taxiing to the ATC, who asked for more information about the aircraft. |
| 5 | Transair 810: “Okay, Rhoades 810, we have lost number one engine and we are coming straight to the airport we are going to need the fire department there is a chance we are going to lose the other engine too. It's running very hot and ah speed is ah we are pretty low on speed. It doesn’t look real good up here, you might want to let the coast guard know as well. And we don’t have any hazmat and ah fuel is about two hours of fuel.  ATC: “And Rhoades Express 810, how many people are on board?” | Transair 810 told the ATC that they had lost their engine and that they are returning to the airport. The ATC sought further details (i.e., souls on board). |
| 6 | United Airlines 1448: “tower this is united fourteen forty eight we are currently on a runway I'm looking out to the right with a kilo uh we need to go on the kileo taxiway.”  ATC: “united fourteen forty eight you were supposed to taxi november and tango I need to know what runway you're on. I can't see anything from the tower.” | United Airlines 1448 mistakenly ended up at the edge of the runway it had just landed on. The aircraft voiced that they were on a runway and the ATC asked what runway they were on. |

#### Elaborating

This type of sensemaking encompasses sharing updates about the situation, problem, or next steps. It is expanding on safety concerns based on the changed situation (e.g., they lost another engine). Elaborating is purely verbal, sharing information about the problem/situation/next steps (actions are ‘implementing’).

##### Inclusions

Expanding on the problem (e.g., ‘lost another engine’), answering questions asked without a change in the course of action (e.g., ‘the left engine is gone’), sharing possible explanations of the problem, sharing information (e.g., weather at the airport, high terrain), sharing ideas for next steps, getting more information (e.g., FedEx 1170 flying over the airport to see if the landing gear was down), giving facts, electronic voices (e.g., ‘CLIMB CLIMB CLIMB’).

##### Exclusions

Asking for repetitions, offering alternative solutions, asking questions, and determining whether someone spoke up (these are all ‘questioning’). Confirming actions (e.g., ‘going around’, ‘stopping take-off’), acknowledgements (e.g., ‘Roger’, ‘Affirm’), new or updated instructions (e.g., ‘climb to 4,000 feet’, ‘go around and follow standard missed approach’), identifying specific individuals who should hear safety voice, answering questions with a change in action (e.g., ‘I’m turning left now’ in response to ‘are you turning left?’) (these are all ‘Implementing’).

# Table 5: Safety listening as elaborating

|  | **Transcript excerpt** | **Interpretation** |
| --- | --- | --- |
| 1 | First officer: “smells like wood burning smell that?”  Second officer: “yeah. I smelled it for a couple of seconds.” | UPS 1307 had cargo smoke, and the first officer raised the concern of smelling a strange odour to the second officer, who smelled it too. They then discussed where the smell was coming from, what it could be, and how to address it. |
| 2 | Crew: “Is [there a] fire? What's going on?”  Crew: “Probably a fire.” | Lot Polish Airlines had an engine fire, and the crew were sensemaking what was happening. Them saying “what’s going on?” and “probably” indicate uncertainty about what the problem was. |
| 3 | Guide: “I think it’ll [i.e., the mountain] be left.”  Flight engineer: “Yes, I reckon about here.” | Air New Zealand 901 was figuring out where they were in relation to a mountain. Them saying “I think” and “I reckon” indicate their uncertainty about where the mountain was. |

## Not engaging with safety voice

Non-engagement with safety voice comprises dismissing, and token listening. These behaviours can be challenging to identify from single dialogue turns and sometimes become apparent when examining subsequent actions and outcomes reported in transcripts and incident reports.

#### Dismissing

Dismissing involved listeners hearing voice but not seriously considering its content. Dismissing can be overt (e.g., defensiveness, retaliation); however, it often is rare and implicit in aviation transcripts. It looks like listeners glossing over concerns or telling voicers to be quiet. Table 6 shows examples.

Ignoring is a lack of behavioural response to safety voice. Listeners may not respond to voice’s content due to distractions (e.g., whether the captain returns from break; rumours; finding the runway; Table 6).

##### Inclusions

Irrelevant responses to speaking up by the intended listener, problems with hearing voice (e.g., broken radio), defensiveness (i.e., avoiding, delegitimizing, limiting), retaliation, rudeness, failing to consider the voiced request, silencing voicers.

##### Exclusions

Considered disagreement with voice which they believe is misguided or infeasible (this would be declining); ATCs asking aircraft if they can hear them (this would be questioning).

# Table 6: Safety listening as dismissing

|  | **Transcript excerpt** | **Interpretation** |
| --- | --- | --- |
| 1 | First officer: “stall warning”  Captain: “I got it, back off.” | In Kalitta 808, the airplane was stalling, and the flight engineer and first officer warned the captain. The captain dismissed the warnings, and the airplane crashed. |
| 2 | ATC: “You are going towards Runway 20.”  Pilot in command: “I think we are going to Runway 02.” | In US-Bangla Airlines 221, the aircraft mistakenly approached Runway 20 despite clearance for Runway 02. The ATC correctly pointed this out and the Pilot in Command dismissed the ATC. The Pilot in Command had lost situation awareness, indicated when he then privately asked the First Officer, “Can we see the Runway? We have set up everything, all done but we are not seeing the Runway”. |
| 3 | United Airlines 1448: “ma'am I'm trying to advise you we're on an active runway united fourteen forty eight.”  ATC: “two three right is not an active runway it's a taxiway when we're i f r or in the dark.” | United Airlines 1448 mistakenly taxied to an active runway in the fog and at night. They were warning the ATC about their position; however, the ATC dismissed their concern, wrongly saying that 23R was not a runway. |
| 4 | United Airlines 1448: “ma'am this is united fourteen forty eight we're on two three right we're looking at kilo straight ahead if we can go straight we can get on kilo and get off the runway.”  ATC: “united fourteen forty eight standby please don't talk I have other things I need to do.” | Later in the same transcript, United Airlines 1448 warned the ATC about their position and suggested how they might leave the runway. The ATC dismissed their concern, telling them not to speak. |
| 5 | First Officer-1: “I’m in TOGA, huh?”  First Officer-2: “Damn it, is he [i.e., the captain] coming or not? We still have the engines! What the hell is happening? I don’t understand what’s happening.” | In Air France 447, ice crystals caused the autopilot to disconnect, resulting in temporary airspeed measurement inconsistencies. Rather than correctly keeping the aircraft at the same altitude, First Officer-1 kept climbing it, leading to a stall. First Officer-1 mentioned that they were climbing the plane (i.e., in Take Off, Go Around) while First Officer-2 focused on the captain returning from his break. |
| 6 | First Officer: “Sir, shouldn’t I switch on the radar?”  Pilot in Command: “That fucker Lamia, she made me… look at my fucking eyes… weeping… crying…” | In US-Bangla Airlines 211, the Pilot in Command had a breakdown mid-flight about a rumour that him and a colleague had sex in the cockpit. The First Officer was trying to fly the aircraft; however, the Pilot in Command was not engaging with her concerns. |
| 7 | Flight engineer: “You know, we’re not gettin’ our airspeed back there.”  Captain: “Where’s the strobe?” | In Kalitta 808, the flight engineer was raising concerns about stalling; however, the captain and the first officer were focused on finding the strobe light identifying the runway. |

#### Token listening

The final type of non-engagement is token listening, where listeners verbally acknowledge safety voice, but their actions indicate a lack of thorough consideration of the voiced content. Token listening is evident in inconsistencies between listeners’ verbal responses and their actions (e.g., saying they are climbing while descending the aircraft), in subsequent conversation turns (e.g., voicers observing that the aircraft was descending), and in outcomes (e.g., hitting the aircraft they supposedly were looking out for). Table 7 shows examples.

##### Inclusions

Pretend engagement with voice acts, as evidenced by additional voice turns or outcomes.

##### Exclusions

Incorrect statements (this would be misguided safety voice or listening).

# Table 7: Safety listening as token listening

|  | **Transcript excerpt** | **Interpretation** |
| --- | --- | --- |
| 1 | ATC: “Descend to 35 [i.e., 3,500’] now.”  South African DC-40: “Descending to 35 now.”  South African DC-40: “We are now 4,000 ft descending.” | The ATC instructed South African DC-10 to descend, and the DC-10 confirmed. Yet, in the next turn, DC-40’s cockpit said they were still at 4,000 feet, indicating that despite confirming the descent order, they did not actually descend. The DC-40 collided with another aircraft at 4,000’ shortly after. |
| 2 | ATC: “climb immediately, maintain 4,000’.”  N7022G: “4,000’, climbing immediately.”  ATC: “Okay, it looks like you’re descending, sir. I need to make sure you are climbing, not descending.” | The ATC told N7022G to climb and the pilot verbally confirmed. However, the pilot descended the plane, highlighting a disconnect. The investigation revealed that the pilot had lost spatial orientation. |
| 3 | ATC: “Additional traffic north shore, it’s a Metroliner for the parallel.”  N416DJ: “I have traffic in sight, cleared to land 17R, 6DJ.” | The ATC warned N416DJ about another aircraft (i.e., Key Lime 970). N416DJ said they saw the traffic; however, they collided mid-air with Key Lime 970 shortly afterwards. |

# Additional safety voice acts

If voicers or third parties sometimes engaged in additional voice acts. These additional voice acts built on the original safety voice, further prompting listeners to act on or sense-make problems. They typically are more direct and explicit than the initial raised concerns. Please code additional safety voice acts in the ‘event’ column in the Excel spreadsheet.

## Escalating safety voice

The first type of additional safety voice is escalation. Escalating voice involves the same voicer repeating their concern, often with increased urgency. Please classify escalating voice if – after safety voice and safety listening – the original voicer raises the same safety concern. It will likely be raised in a clearer and more direct fashion.

For example, the ATC engaged in escalating voice by repeatedly instructing Air China 428 to turn right after receiving no response to their initial warnings (Excerpt 1, line 19). This escalation helped the crew understand the imminent collision risk with the mountains, leading to a shared situation awareness and corrective action.

##### Inclusions

The same human voicer raising the same safety concern after non-engagement.

##### Exclusions

Expanding on safety concerns based on the changed situation (e.g., they lost another engine) if the original voicer is the speaker (this is ‘elaborating’). Electronic voices (this is ‘elaborating’). Raising the same concern after engagement (this is ‘elaborating’).

# Excerpt 1: Escalating safety voice (Air China 428)

| In 2017, Air China 428 (‘CCA428’) incorrectly turned left heading towards mountains upon departure from Hong Kong International Airport. Here, the ATC instructs CCA428 to turn right immediately and expedite climb, averting an accident. | | | | |
| --- | --- | --- | --- | --- |
| **Line** | **Speaker** | **Transcript** | **Code** | **Interpretation** |
| 16 | ATC | Air China 428, turn right immediately. Turn right immediately. Heading 0 – correction – heading 270. Terrain ahead. Expedite climb. | Safety voice | The ATC told CCA428 to turn right and climb immediately as there was terrain ahead. |
| 17 | ATC | Air China 428? | Escalating safety voice | No response from CCA428; the ATC checked if they have heard. |
| 18 | CCA428 | [Radio noises] |  | Unclear response from CCA428. |
| 19 | ATC | Air China 428, expedite climb. Terrain ahead – terrain alert! Expedite passing 5000 feet. Expedite! | Escalating safety voice |  |
| 20 | CCA428 | Expedite, Air China 428. | Implementing |  |

## Amplifying safety voice

Amplifying voice involves a third party (i.e., not the original voicer) reiterating the safety message. Amplifying voice played a critical role in averting potential accidents by reinforcing concerns and prompting necessary actions after initial non-engagement. In Air Canada 759 (Excerpt 2, line 7), United 1, an aircraft on the taxiway, noticed that Air Canada 759 was aligned with the taxiway rather than the runway. Amplifying voice reinforced the Air Canada cockpit’s muted concern about seeing lights, helping to avoid a multi-aircraft collision.

##### Inclusions

Third party amplifying the original safety voice’s message.

##### Exclusions

Expanding on safety concerns based on the changed situation (e.g., they lost another engine) if someone other than the original voicer is the speaker (this is now ‘elaborating’). Electronic voices (this is ‘elaborating’). Raising the same concern after engagement (this is ‘elaborating’).

# Excerpt 2: Amplifying safety voice (Air Canada 759)

| In 2017, Air Canada 759 (‘ACA759’) accidentally lined up for landing on taxiway C (containing four aircraft) instead of runway 28R. United Airlines Flight 1 (‘UAL1’) communicated this error on the open channel and the ATC told ACA759 to abort landing. | | | | |
| --- | --- | --- | --- | --- |
| **Line** | **Speaker** | **Transcript** | **Code** | **Interpretation** |
| 4 | ACA759 | And Tower, just wanna confirm – it’s Air Canada 759, we see some lights on the runway there. Please, confirm we are cleared to land? | Safety voice | ACA759 said that they saw unusual lights on the runway. |
| 5 | ATC | Air Canada 759, confirm. Cleared to land runway 28R. There is no one on 28R but you. | Elaborating | ATC did not acknowledge the lights seen by ACA759. |
| 6 | ACA759 | Okay, Air Canada 759. | Implementing | ACA759 confirmed the information. This confirmation resulted in a shared misperception that there was no problem. |
| 7 | UAL1 | Where’s this guy going? He’s on the taxiway! | Amplifying safety voice | UAL1 told the open channel that ACA759 was landing on taxiway C. This safety voice strengthened ACA759’s initial attempt. |
| 8 | ATC | Air Canada, go around. | Implementing | The ATC told ACA759 to abort landing. |
| 9 | ACA759 | In the go around, Air Canada 759. | Implementing | ACA759 confirmed that they have aborted landing. |

# References

Noort, M.C., Reader, T.W., Gillespie, A., 2019. Speaking up to prevent harm: A systematic review of the safety voice literature. Saf. Sci. 117, 375–387. https://doi.org/10.1016/j.ssci.2019.04.039

Pandolfo, A.M., Reader, T.W., Gillespie, A., 2024. Safety listening in organizations: An integrated conceptual review. Organ. Psychol. Rev. 15. https://doi.org/10.1177/20413866241245276
